# Defining unique structural features in the MAFA and MAFB transcription factors that control *Insulin* gene activity

**DOI:** 10.1016/j.jbc.2024.107938

**Published:** 2024-10-28

**Authors:** Jeeyeon Cha, Xin Tong, Katie C. Coate, Min Guo, Jin-hua Liu, Garrett Reynolds, Emily M. Walker, Richard A. Stein, Hassane Mchaourab, Roland Stein

**Affiliations:** 1 Department of Molecular Physiology & Biophysics, Vanderbilt University, Nashville, Tennessee, USA; 2 Division of Diabetes, Endocrinology, and Metabolism, Department of Medicine, Vanderbilt University Medical Center, Nashville, Tennessee, USA; 3 VA Tennessee Valley Healthcare System, Nashville, Tennessee, USA; 4 Division of Metabolism, Endocrinology & Diabetes, Departments of Molecular & Integrative Physiology and Internal Medicine, University of Michigan, Ann Arbor, Michigan, USA; 5 Center for Applied Artificial Intelligence in Protein Dynamics, Vanderbilt University, Nashville, Tennessee, USA

**Keywords:** transcription factors, MAFA, MAFB, *Insulin*, islet biology, AlphaFold 2, gene expression, transactivation

## Abstract

MAFA and MAFB are related basic-leucine-zipper domain-containing transcription factors which have important overlapping and distinct regulatory roles in a variety of cellular contexts, including hormone production in pancreatic islet cells. Here, we first examined how mutating conserved MAF protein-DNA contact sites obtained from X-ray crystal structure analysis impacted their DNA-binding and *Insulin* enhancer-driven activity. While most of these interactions were essential and their disruption severely compromised activity, we identified that regions outside of these contact sites also contributed to transcriptional activity. AlphaFold 2 (https://alphafold.ebi.ac.uk), an artificial intelligence-based structural prediction program, was used to determine if there were also differences in the three-dimensional organization of the non-DNA binding/dimerization sequences of MAFA and MAFB. This analysis was conducted on the WT proteins as well as the pathogenic MAFA^Ser64Phe^ and MAFB^Ser70Ala^ transactivation domain mutants, with differences revealed between MAFA^WT^ and MAFB^WT^ as well as between MAFA^Ser64Phe^ and MAFA^WT^, but not between MAFB^Ser70Ala^ and MAFB^WT^. Moreover, dissimilarities between these proteins were also observed in their ability to cooperatively stimulate *Insulin* enhancer-driven activity in the presence of other islet-enriched transcription factors. Analysis of MAFA and MAFB chimeras disclosed that these properties were influenced by their unique C-terminal region structural differences predicted by AlphaFold 2. Our findings have revealed key structural features of these closely related proteins that impact their ability to regulate gene expression.

The musculoaponeurotic fibrosarcoma (MAF) family consists of protooncogenes with important cellular regulatory properties that can be categorized into two groups: small MAFs that lack an N-terminal transactivation domain (∼150–160 amino acids: MAFF, MAFG, and MAFK) and large MAFs (∼240–340 amino acids: MAFA, MAFB, c-MAF, and NRL) (1, 2). These MAF proteins act as transcription factors (TFs) and play active roles in many tissue types, such as the pancreas (3, 4), lens of the eye (4), and cartilage (5) by contributing to their development, differentiation, and regulation of mature function.

The MAFA and MAFB proteins have also been found to be vital in the development and/or maturation of pancreatic islet *Insulin* hormone-producing β- and glucagon hormone-producing α-cells (1, 2). Expression of these proteins is affected in type 1 and type 2 diabetes (6, 7, 8, 9), and mutations near a priming phosphorylation site in both MAFA and MAFB are clinically relevant. The Ser64Phe (*i.e.*, S64F) substitution prevents the priming serine (S) 65 phosphorylation of MAFA and produces either monogenic diabetes or insulinomatosis (10). The Ser70Ala (*i.e.*, S70A) mutation in MAFB blocks serine (S) 70 phosphorylation and causes multicentric carpotarsal osteolysis (*i.e.*, MCTO), a pediatric multisystem disorder characterized by osteolysis of the carpal and tarsal bones, subtle craniofacial deformities, and nephropathy (11).

The X-ray crystal structure has been determined for the basic-leucine-zipper regions of MAFA (12) and MAFB (13), which are responsible for DNA binding and homodimerization or heterodimerization (14). Here, we first analyzed how mutations in conserved basic region amino acids which interacted with DNA control element sequences in these crystallographic studies influenced *Insulin* enhancer-driven and DNA-binding activity of the full-length protein. While most of these interactions were critical, one variant of MAFA Arg272Ala (MAFA^R272A^) functioned similarly to the WT protein while its analogous mutation in MAFB (MAFB^R256A^) was debilitating, suggesting contributions from regions lying outside of the basic-leucine-zipper domains. To further analyze these areas, we applied AlphaFold 2 algorithm to obtain insight into their underdefined N-terminal transactivation domain and C terminal region structure. Functional and molecular assays were used to evaluate the relevance of the predicted structural differences to the activity of MAFA^WT^, MAFB^WT^ and their pathogenic variants. Our results strongly suggest that structural dissimilarities between MAFA and MAFB impart their unique functional properties under physiologic and disease conditions.

## Results

### In contrast to MAFB^R256A^, the analogous MAFA^R272A^ mutant does not impact transactivation or DNA binding activity

**Figure 1:**
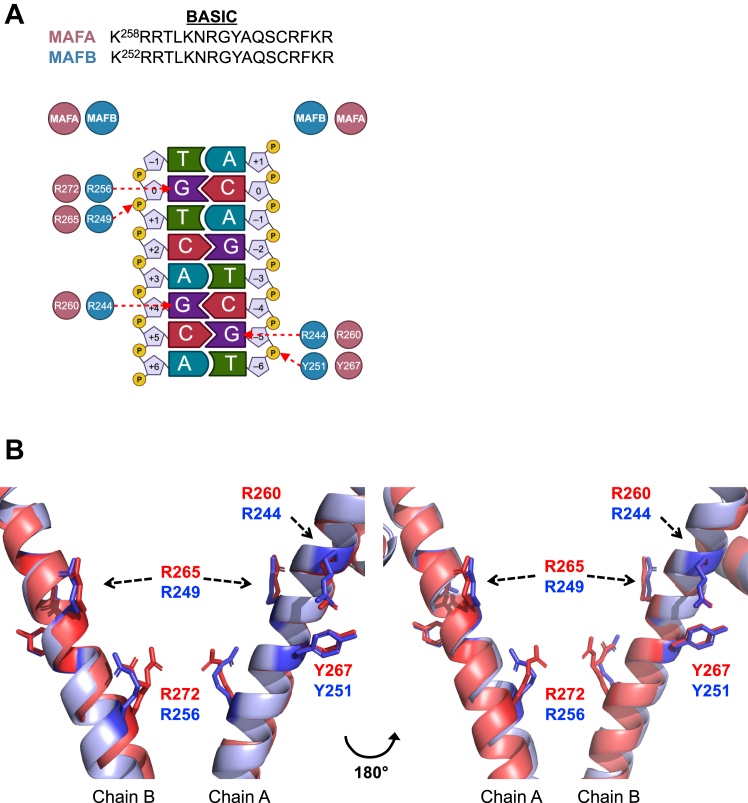
**X-ray crystal structure analysis revealed that conserved basic region—DNA binding MAFA**^**R272**^**has a unique rotameric structure to the analogous MAFB**^**R256**^. *A*, *Top*: the sequences of basic region are identical between MAFA, MAFB, and the other closely related large MAF proteins (*i.e.*, c-MAF and NRL (12). *Bottom*: schematic representation of X-ray crystallographic results illustrating the MAFA and MAFB basic region amino acid base-specific interactions under analysis. *B*, opposite views of the DNA overlay of the crystal structures for MAFA (4EOT, *red*) (19) and MAFB (2WTY, *blue*) (12). The four residues shown in stick schematics are R260, R265, Y267, and R272 of MAFA and R244, R249, Y251, and R256 of MAFB. MAF, musculoaponeurotic fibrosarcoma.

The amino acids comprising the basic region of MAFA and MAFB are absolutely conserved as were *cis*-control element DNA contacts within the *Escherichia* *coli* expressed basic leucine-zipper portion of the protein (*i.e.*, MAFA, residues 226–318; MAFB, 211–323) analyzed previously by X-ray crystallography (Fig. 1*A*) (12, 13). We first determined how mutation of four amino acids within their basic region would affect DNA-binding and transstimulation activity of full-length MAFA and MAFB, considering the possibility that posttranslational events (*e.g.*, phosphorylation (15), ubiquitination (16), acetylation (17) and sumoylation (18)) produced within these heavily modified proteins in mammalian cells could be influential. Of note, the X-ray crystal structure revealed that three of the analyzed residues in MAFA (*i.e.*, R260, R265, and Y267) and MAFB (R244, R249, and Y251) have essentially the same rotameric alignment in relation to the *cis*-acting control element DNA sequences and have near-complete overlap in chains A and B of the protein homodimer (Fig. 1*B*). In contrast, even though MAFA^R272^ and MAFB^R256^ contact the same control element guanine, they used a distinct rotameric state for binding (12, 19) (Fig. S1).

**Figure 2:**
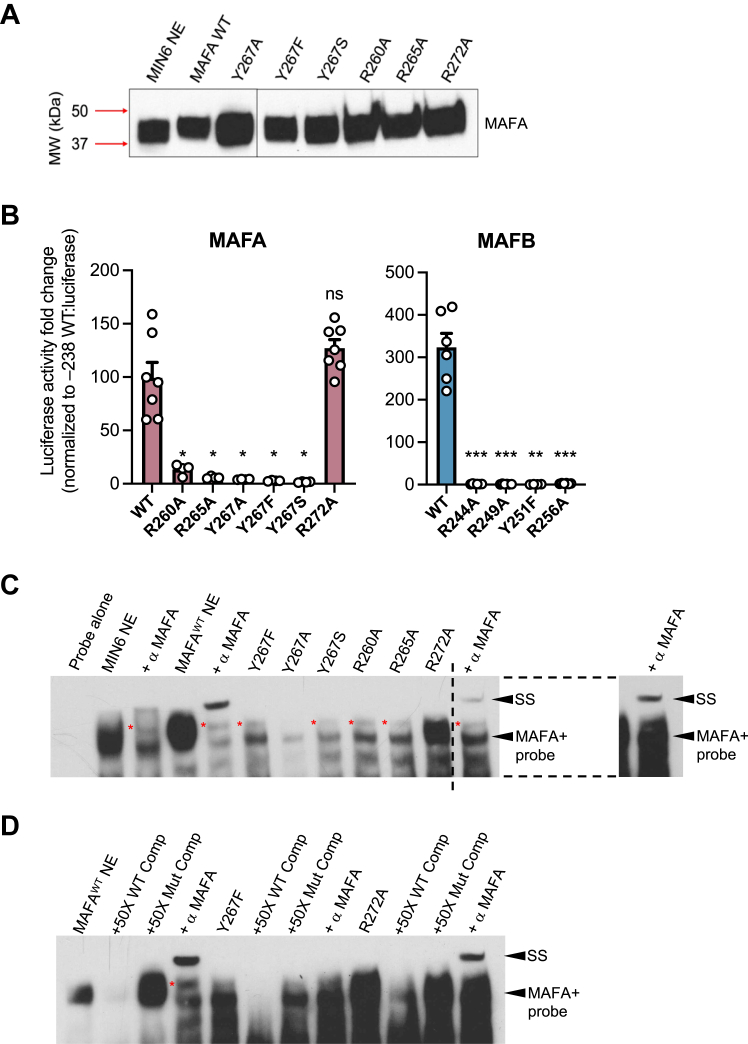
***Insulin* enhancer and DNA binding activity is retained in MAFA**^**R272A**^**, but not other conserved MAFA or MAFB basic regions identified in crystallographic DNA binding analysis.***A*, MAFA full-length mutant protein were produced at WT-like levels (MAFA, Bethyl Laboratories, Product # A700–067). *B*, WT and mutant MAFA (*left*) and MAFB (*right*) expression vectors were transiently transfected into HeLa cells with the *Insulin* −238 WT reporter. All error bars indicate SEM. ∗∗∗*p* < 0.001; ∗∗*p* < 0.01; ∗*p* < 0.05. n = 3–4. *C*, gel shift assays of HeLa nuclear extract (NE) produced WT and mutant MAFA bound to *INS* enhancer. MIN6 NE served as a positive control extract. Only MAFA^WT^ and MAFA^R272A^ were super-shifted (SS) upon addition of MAFA antibody. Right, increased exposure to show SS of MAFA^R272A^. *Dashed lines* signify where image was cropped for increased exposure. *Red asterisk*, nonspecific band. *D*, MAFA^Y267F^ competed effectively with mutant oligo but could not super-shifted with MAFA antibody, whereas both MAFA^WT^ and MAFA^R272A^ did. *Red asterisk*, nonspecific band. MAF, musculoaponeurotic fibrosarcoma; INS, *Insulin*.

The three conserved arginines (*i.e.*, R) and the unique tyrosine (Y) of the MAFA and MAFB basic region were mutated to alanine (A) and phenylalanine (P), respectively. The full length WT and mutant proteins were produced in transfected HeLa cells and analyzed for their ability to stimulate *Insulin*-enhancer driven reporter activity, bind *Insulin* control element DNA in gel shift assays, and stably produce protein by immunoblotting. All of the mutants were expressed at similar levels (Figs. 2*A*, S2), and each of the basic region DNA-binding mediating mutants of MAFB (*i.e.* R244A, R249A, Y251F, and R256A) and most in MAFA (R260A, R265A, and Y267A/F/S; Fig. 2*B*) were debilitating in the reporter assays. MAF^R272A^ had WT-like activity whereas the comparable MAFB^R256A^ mutant was inactive (Fig. 2, *B* and *C*). Notably, the MafA antibody super-shifted MAFA^R272A^ (albeit with lower efficiency than MAFA^WT^ in this assay) and competed specifically in gel shift assays, whereas the dysfunctional mutants did not (Fig. 2*D*). The results indicated that features in MAFA and MAFB residing outside the region analyzed earlier (12, 13) may be consequential to activity.

### AlphaFold 2 predicts structurally unique regions within the MAFA, MAFB, and their pathogenic variant proteins

**Figure 3:**
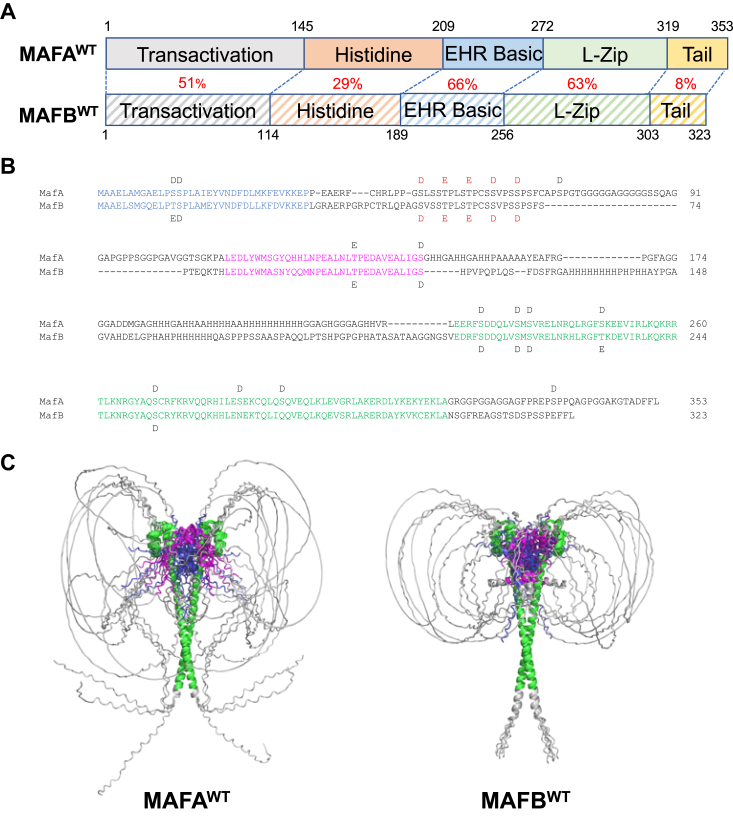
**AlphaFold 2 modeling of MAFA**^**WT**^**and MAFB**^**WT**^**protein.***A*, protein sequence identity (%) between various domains of MAFA^WT^ and MAFB^WT^ are denoted in *red*. Extended homology domain (EHR); Leucine-zipper (L-Zip). *B*, the aligned amino acid sequences of MAFA^WT^ and MAFB^WT^, with aspartic acid (*D*) and glutamic acid (*E*) used as phosphomimetics at *bona fide* sites of phosphorylation (15). The *red* labeled *E* and *D* represent the phosphosites absent in the underphosphorylated variants MAFA^S64F^ and MAFB^S70A^. The regions marked in *blue* (*i.e.*, 83%) and *magenta* (*i.e.*, 79%) have higher sequence conservation than the overall transactivation domain and have structural elements in the AlphaFold 2 models. The region in *green* is the basic-leucine-zipper domain structured by AlphaFold 2 and determined by crystallography (*i.e.*, MAFA residues 226–318 and MAFB residues 211–302) (12, 13). *C*, comparing the five independently derived representative models for MAFA^WT^ (*left*) and MAFB^WT^ (*right*) illustrates potential structural difference in many areas of these proteins. For example, the longer C terminal tail of MAFA^WT^ leads to a more divergent conformation than the MAFB^WT^ that may interact with N terminal region sequences. MAF, musculoaponeurotic fibrosarcoma.

The level of sequence identity between MAFA and MAFB is variable between domains: the N-terminal transactivation domain (51%), histidine-rich region (29%), extended homology region (66%), basic (100%), leucine-zipper dimerization region (63%), and C terminal tail (8%) (2) (Fig. 3*A*). Structural information outside the basic-leucine-zipper region of MAFA and MAFB is likely of importance, as for example, preventing priming phosphorylation at S65 in MAFA and subsequently GSK3-mediated phosphorylation at serine (S) 61, threonine (T) 57, T53, and S49 unpredictably, and significantly changes SDS-PAGE mobility (*i.e.* 46–42 kD (16, 20). In addition, the MAFA DNA-binding ability, but not MAFB, appears to be regulated by phosphorylation within the N-terminal transactivation domain region (15).

MAFA and MAFB act as homodimers or heterodimers under physiological conditions (21). Here, we used AlphaFold 2 for *de novo* modeling of homodimers of full-length MAFA and MAFB. AlphaFold 2 uses deep iterative learning to provide predictions of the three-dimensional architecture of a protein based on multiple sequence alignments (22). The analysis was performed on unphosphorylated MAFA (MAFA^un^) and MAFB (MAFB^un^), fully phosphorylated MAFA (MAFA^WT^), and MAFB (MAFB^WT^), and partially phosphorylated mutant MAFA (MAFA^S64F^) and MAFB (MAFB^S70A^). MAFA^un^ (and presumably MAFB^un^) is unable to bind DNA (15). For the purpose of modeling, aspartic (D) and glutamic (E) acid were used as phosphomimetics at S and T, respectively (Fig. 3*B*). MAFA^S64F^ and MAFB^S70A^ have a reduced phosphorylation state relative to the WT protein due to the inability of a yet unidentified priming kinase to act at S65 in MAFA^S64F^ or S70 in MAFB^S70A^ (Fig. S3*A*).

Using a locally installed version of AlphaFold 2, we obtained 25 intrinsic structural models of the MAFA^WT^ and MAFB^WT^ from five seeds, allowing for a more robust exploration of conformational ensembles. The models for each of the sequences yields dimers with the expected folds within the basic-leucine-zipper domains, while the transactivation domain had no specific conformation (Fig. 3*C*). We noted differences in the nonconserved C terminal tail between MAFA and MAFB as well as the highly variable loops comprising the histidine-rich regions and the center of the transactivation domain. The amino and carboxy termini of the *trans*-activation domain in the models display some secondary structure. Although there is no consensus on their absolute placement, they invariably lie near the DNA binding region of the basic-leucine-zipper domain (Fig. 3*C*).

Comparison of MAFA^WT^ to MAFA^un^ or MAFB^WT^ to MAFB^un^ indicates that the phosphomimetics result in a change of the AlphaFold 2 predicted models (Fig. S3, *B* and *C*). Moreover, reducing the extent of phosphorylation in MAFA^S64F^ in comparison to MAFA^WT^ leads to further alterations in the placement of the transactivation domain relative to the leucine zipper, which is depicted upon measuring the distances from Cα in each amino acid residue to a fixed point in space (Fig. S3*D*). The pattern is slightly different in MAFB as the reduction in MAFB^S70A^ phosphorylation does not lead to a predicted change in the location of the transactivation domain (Fig. S3, *C* and *D*). Comparison of MAFA^WT^ to MAFB^WT^ indicates small differences in the regions proximal to the leucine-zipper domain and more substantial differences in the unstructured transactivation domains. However, the most striking change between MAFA^WT^ and MAFA^S64F^ or between MAFB^WT^ and MAFB^S70A^ is in their C terminal region (Fig. S3, *B* and *C*), which is much more disordered in MAFA^WT^ and MAFA^S64F^ and in some models is found near the transactivation domain. Comparisons of these models also yielded the following values RMSD values measuring the similarity between paired structures: MAFA^un^ *versus* MAFA^WT^, 0.740; MAFA^un^ *versus* MAFA^S64F^, 0.680; MAFA^WT^ *versus* MAFA^S64F^, 0.123; MAFB^un^ *versus* MAFB^WT^, 0.784; MAFB^un^ *versus* MAFB^S70A^, 0.807; MAFA^WT^ *versus* MAFA^S70A^, 0.160. In line with the distance measurements, RMSD analysis suggests that the WT and mutant models are more similar to each other than to the unphosphorylated MAFA and MAFB forms.

We also found that the dimerization ability of the mutant proteins is retained by AlphaFold 2; however, the core structure for the heterodimer adopted more than one conformation compared to the single conformation of the homodimer (Fig. S4*A*). Differences in the both the N and C termini in the heterodimer were greater compared to the homodimer and between the different phosphorylation states (Fig. S4*B*). We then sought to determine how these possible structural differences relate to MAFA and MAFB activity.

### WT and mutant MAFA and MAFB differ in their ability to functionally interact with other TFs

**Figure 4:**
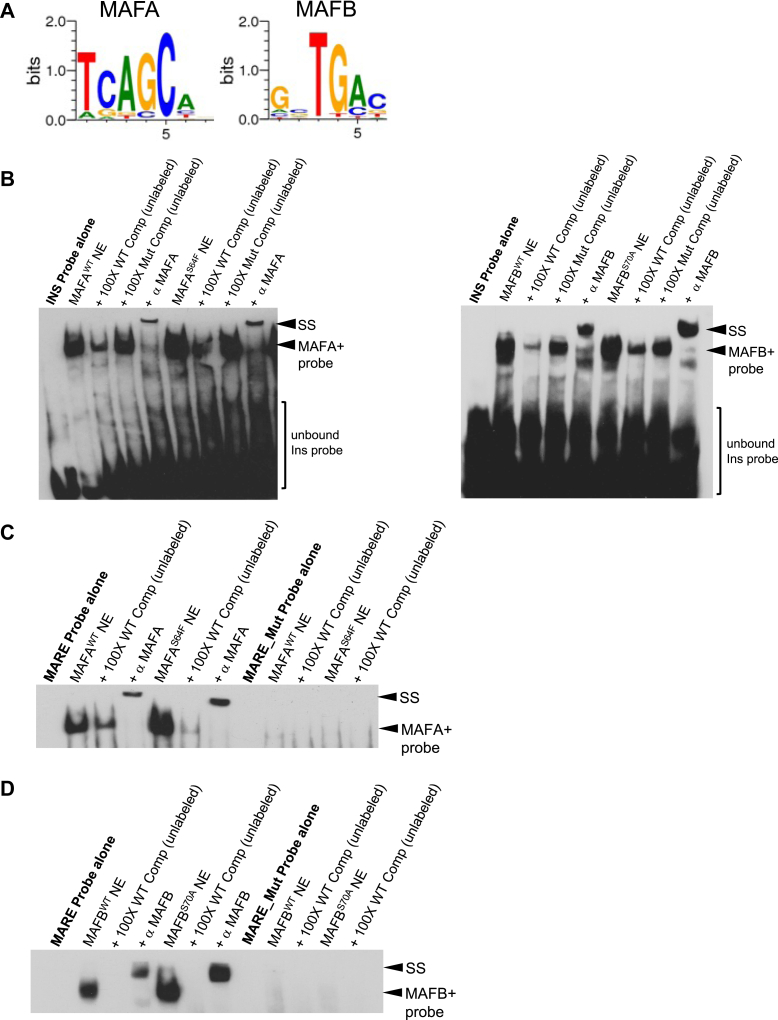
**Comparable binding affinity of MAFA, MAFB and their pathogenic mutants to known target sequences.***A*, the DNA binding sequence motif of MAFA and MAFB (34) as determined by ChIP-seq databases. *B*, gel shift assays of HeLa nuclear extract producing WT and mutant MAF protein effectively bound to *INS* enhancer. Specificity of binding was interrogated by unlabeled competitor probes in excess. MAFA^WT^ and MAFA^S64F^ were then super-shifted (SS) upon addition of MAFA antibody, while MAFB^WT^ and MAFB^S70A^ were super-shifted (SS) upon addition of MAFB antibody. *C* and *D*, gel shift assays of HeLa nuclear extract producing WT and mutant MAF proteins effectively bound to the MARE consensus sequence. Specificity of binding was interrogated by unlabeled competitor probes in excess. MAFA^WT^ and MAFA^S64F^ were super-shifted (SS) upon addition of MAFA antibody, while MAFB^WT^ and MAFB^S70A^ were super-shifted (SS) upon addition of MAFB antibody. MAF, musculoaponeurotic fibrosarcoma; INS, *Insulin*.

MAFA and MAFB, like many other TFs are important in cell development and adult function, exhibit cell and temporal specificity in expression and act in concert with other TFs by binding and regulating enhancer activity (23). MAFA and MAFB are uniquely coexpressed only in pancreatic islets, skeletal muscle, and testes, and these proteins bind to closely related *cis*-acting element sequences (Fig. 4*A*). For example, in the pancreatic islet, both MAFA and MAFB are important in *Insulin (INS)* gene transcription across species (1, 24). We first compared the binding activity of MAFA and MAFB to the *INS* enhancer with that of their pathogenic mutants and found similar binding capacity (Fig. 4*B*). We also compared their abilities to bind to the MAF consensus binding sequence (MARE), which were also similar, and binding was similarly impaired between the WT and variant proteins using a nonspecific binding probe, MARE_Mut (Fig. 4, *C* and *D*). Mutating the 5′ flanking TGC sequence of the MARE dramatically increased the dissociation rate of the probe to the WT or pathogenic MAF proteins, while disrupting the central C_0_ of the MARE sequence did not alter this interaction (Fig. S5) (12). Thus, predicted structural differences did not appear to impact direct binding capacity to a known target sequence.

**Figure 5:**
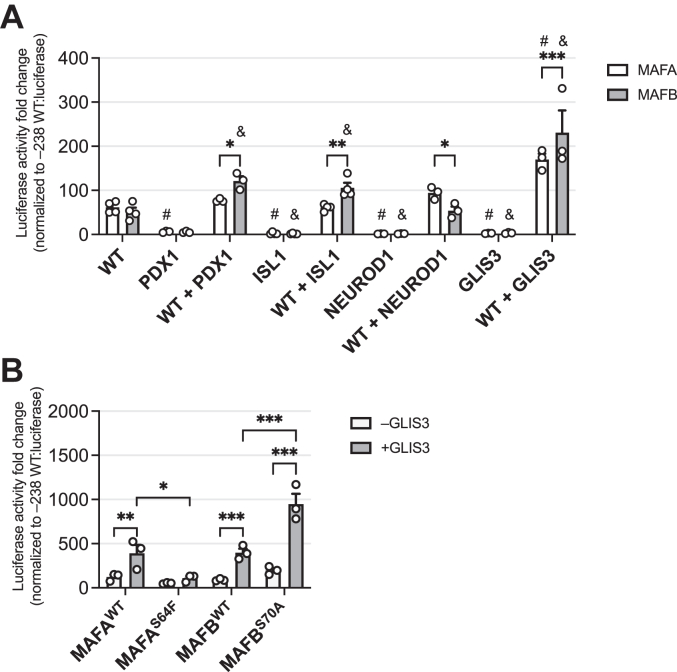
**Comparing the ability of WT and mutant forms of MAFA and MAFB to act with other islet-enriched TFs to stimulate*****Insulin*****-driven reporter activity.***A*, dual luciferase reporter assays were performed in HeLa cells transfected with vectors producing MAFA^WT^ (*white bars*), MAFB^WT^ (*gray bars*), PDX1, ISL1, NEUROD1, and/or GLIS3. The fold-change in activity of the −238 WT reporter over the no TF transfected controls is shown. Two-way ANOVA with Dunnett’s multiple comparisons test was performed. All error bars indicate SEM. Between group comparisons: ∗∗∗*p* < 0.001; ∗∗*p* < 0.01, ∗*p* < 0.05. Within group comparisons: ^#^*p* < 0.05 vs WT MAFA, ^&^*p* < 0.05 vs WT MAFB. n = 3–4. B, Dual luciferase reporter assays were performed in HeLa cells transfected with vectors that express MAFA^WT^, MAFB^WT^, MAFA^S64F^, and MAFB^S70A^ with or without GLIS3. The fold-change in activity of the −238 WT reporter over the no TF transfected control is shown. Two-way ANOVA with Tukey’s multiple comparisons test was performed. All error bars indicate SEM. ∗∗∗*p* < 0.001, ∗∗*p* < 0.01; ∗*p* < 0.05. n = 4. MAF, musculoaponeurotic fibrosarcoma; TF, transcription factor.

To determine whether MAFA^WT^ and MAFB^WT^ differentially impact cooperative activation by other islet-enriched TFs which can also bind to the *Insulin* gene, these proteins were cotransfected with PDX1, NEUROD1, ISL1, and GLIS3 and analyzed for their ability to increase *Insulin*-driven −238 WT:luciferase reporter activity in HeLa cells which do not express these factors at detectable levels (Fig. S6*A*). Notably, only the MAFA and MAFB proteins were able to independently stimulate *Insulin*-reporter activity (Fig. 5*A*). MAFB, but not MAFA, provided additional enhanced activation in the presence of PDX1 and ISL1, while both MAFA and MAFB functioned cooperatively with GLIS3. In contrast, NEUROD1 did not elevate MAFA^WT^ or MAFB^WT^ activity, although the protein was expressed effectively (Fig. S6*B*). Differences were also observed in the ability of MAFA^S64F^ and MAFB^S70A^ to functionally interact with GLIS3, as MAFB^S70A^ provided additional stimulation over MAFB^WT^, whereas this enhancement was lost in MAFA^S64F^ (Fig. 5*B*). Collectively, these results suggested that structural differences between the MAFA and MAFB proteins influence their ability to functionally interact with other islet-enriched TFs.

### Analyzing how the predicted structurally unique regions of MAFA and MAFB affect ISL1 and PDX1 activation

**Figure 6:**
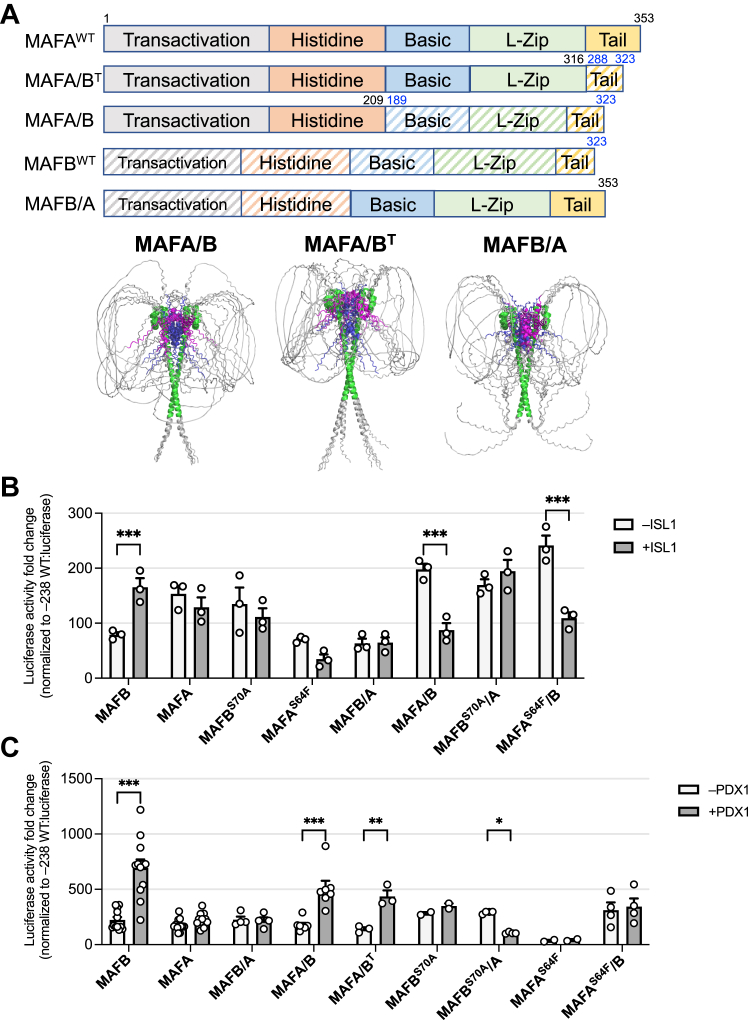
**ISL1 does not activate the MAFA/B and MAFB/A chimeras whereas PDX1 activation requires MAFB C-terminal sequences.***A*, *Top*: the regions of the MAFA and MAFB chimeric proteins. Bottom: The models for the MAFA/B (*left*), MAFA/B^T^ (*center*), and MAFB/A (*right*) chimeras. *B*, dual luciferase reporter assays were performed in HeLa cells transfected with vectors that express MAFB^WT^, MAFA^WT^, MAFB^S70A^, MAFA^S64F^, MAFB/A, MAFA/B, MAFB^S70A^/A, MAFA^S64F^/B, and/or ISL1. Two-way ANOVA with Tukey’s multiple comparisons test was performed. All error bars indicate SEM. ∗∗∗*p* < 0.001, ∗∗*p* < 0.01; ∗*p* < 0.05. n = 4. *C*, dual luciferase reporter assays were performed in HeLa cells transfected with vectors that express MAFB^WT^, MAFA^WT^, MAFB/A, MAFA/B, MAFA/B^T^, MAFB^S70A^, MAFB^S70A^/A, MAFA^S64F^, and MAFA^S64F^/B with or without PDX1. Two-way ANOVA with Turkey’s multiple comparisons test was performed. All error bars indicate SEM. ∗∗∗*p* < 0.001, ∗∗*p* < 0.01; ∗*p* < 0.05. n = 4. MAF, musculoaponeurotic fibrosarcoma.

Chimeric proteins between MAFA and MAFB were generated to evaluate how their N- and C terminal regions influenced ISL1 and PDX1 activation of the *Insulin* −238 WT reporter. Importantly, the chimeric proteins retained the unique three-dimensional properties predicted by AlphaFold 2 in the corresponding domains of the WT protein (Figs. 6*A* and S7). Interestingly, ISL1 stimulation was lost in MAFB^WT^ upon exchanging either MAFA^WT^ or MAFA^S64F^ N terminal (*i.e.*, termed MAFA/B wherein amino acids 1–208 of MAFA were linked to residues 189–323 of MAFB^WT^) or C terminal sequences (*i.e.*, MAFB/A) (Fig. 6*B*). These results indicated that the structural integrity of both the N and C terminal regions of MAFB were necessary for cooperative activation with ISL1.

In contrast to ISL1, MAFB^WT^ plus PDX1 activity was retained in N-terminal MAFA/B fusions (Fig. 6*C*). Interestingly, the MAFA/B^T^ chimera that merely retained 35 C-terminal amino acids of MAFB^WT^ also appeared to work together with PDX1, although basal activity was reduced in the MAFB C terminal fusions (*i.e.*, MAFA/B^T^ by ∼84% and MAFA/B by ∼54%). Conversely, PDX1 plus MAFA/B activity was lost in MAFA^S64F^/B. In addition, stimulation with PDX1 was not observed in MAFB/A and even reduced by MAFB^S70A^/A. Collectively, these results imply that the distinct structural features within full-length MAFA and MAFB mediate key functional interactions with other essential islet-enriched TFs.

## Discussion

MAFA^WT^ and MAFB^WT^ play crucial roles in a variety of cell types across development and postnatal life (21). In this study, we first analyzed how mutants constructed within crystallographically determined basic region-DNA control element contact sites impacted protein activity. Notably, MAFA^R272A^ retained WT-like transactivation and DNA-binding activity while the analogous MAFB^R256A^ mutant was inactive. These results implied that sequences residing outside the structurally analyzed basic-leucine-zipper region of MAFA and MAFB were functionally important. Consequently, the deep learning AlphaFold 2 algorithm was used to predict the three-dimensional architecture of the disordered regions within the non-DNA binding/dimerization regions of the WT and a MAFA or MAFB transactivation domain mutant protein, and then analyzed for functional impact. Predicted differences in the N terminal and C terminal disordered regions of MAFA^WT^, MAFA^S64F^, and MAFB^WT^ were found to regulate *Insulin*, a known target gene, in cooperation with islet-enriched PDX1, ISL1, and GLIS3 TFs. Overall, we believe that the newly defined structural regions identified are essential to MAFA and MAFB activity.

The mutations identified within the basic region by X-ray crystallography would be expected to compromise TF DNA-binding ability. This expected property was found for all of the full-length MAFB mutants analyzed and for all but one in MAFA: MAFA^R272A^. It is not entirely clear why this discrepancy occurred in MAFA, but may reflect that the earlier structural analysis was performed on only a portion of an unmodified *E. coli* expressed protein (*i.e.*, aa 226–318 (12)), appreciating that MAFA^WT^ and MAFB^WT^ are normally heavily modified in mammalian cells (*e.g.*, phosphorylation (15), ubiquitination (16), acetylation (17), and sumoylation (18)). This likely includes the novel structural features detected by AlphaFold 2 in the full-length phosphomimetic substituted proteins (Fig. 3*B*), presumably eliminating the expected role of MAFA^R272^ in DNA-binding.

The C terminal tail structure of MAFA and MAFB predicted by AlphaFold 2 had the most striking impact experimentally. Thus, ISL1 activation by MAFB of the *INS*-driven reporter was prevented in the MAFB/A chimera (Fig. 6*B*), while the MAFB^WT^ C terminal conveyed PDX1 activation to MAFA/B and MAFA/B^T^ mutants and compromised basal MAFA^WT^ transactivation activity (Fig. 6*C*). Earlier studies had also revealed novel regulatory properties for the MAFA C terminal tail: ectopic expression of MAFA, but not MAFB, promoted *Insulin*^+^ cell formation within embryonic endoderm in chick *in ovo* electroporation assays (25). Moreover, only the chimeric MAFB/A protein induced chick *Insulin* activation and not a MAFA/B fusion (25). The inability of MAFB to stimulate *Insulin* expression in the *in ovo* electroporation assay suggests that cooperating factors mediating activation during mouse (25) and human (24) pancreatic development are lacking in chick endoderm.

The analysis of PDX1, NEUROD1, ISL1, and GLIS3 on MAFA^WT^ and MAFB^WT^ activity was designed to glean whether their structurally distinct disordered regions predicted by AlphaFold 2 were regulatory. While it is presently unclear how much change DNA-binding would have imparted to these predictions, we believe that the functional interactions between PDX1, ISL1, and GLIS3 with the WT and chimeric proteins reveals their relevance to, at least, *INS*-driven expression. Consequently, we propose that collaboration between TFs like those analyzed here are important in regulating MAFA^WT^ and MAFB^WT^ controlled enhancers *in vivo*, many of which will be in common and some distinct. Our results demonstrated that such regulation entails recruitment of shared (*e.g.*, GLIS3) and dissimilar (*e.g.*, ISL1 by MAFB) TFs.

The primary purpose of our study was to evaluate the relationship between the AI-predicted, full length structures of targeted MAF mutants and their function to ultimately correlate DNA binding capacity (gel shift assays) with activity (transactivation by luciferase reporter) and structure (AlphaFold 2). We find that clinically pathogenic mutants affect activity, and we identify a relationship between MAF protein structures outside of the DNA interacting domain and their abilities to transactivate. Interrogating the impact of MAFA and MAFB at the resolution of specific targeted residues would be exceedingly difficult to perform and analyze outside of a cell culture system. Although stable integration of these factors in systems in which they are endogenously expressed such as β cell lines or pancreatic islets would be valuable, normalization and interpretation of the studies would be challenging since the levels of endogenous MAF factors are variable across donors, islets, and even β cell subpopulations. We chose to employ HeLa cells expressing CMV-driven MAFA, MAFB, PDX1, GLIS3, ISL1, and/or NEUROD1 due to their relative lack of detectable expression of islet-enriched TFs to avoid interference from an endogenous source (Fig. S6*A*). Under these controlled conditions, we found that binding of the mutant MAF proteins to highly evolutionarily conserved enhancer sequences (*i.e. INS*) or the consensus MARE sequence is largely intact (Fig. 4, *C* and *D*), leading us to conclude that differences in transactivation potential are unlikely due to differential target sequence affinity. In fact, cooperative activation of other islet-enriched TF’s is impacted, and the differences in the recruitment of other coregulators and their interactions with non-DNA binding portions of the proteins will be the subject of future analysis.

It is noteworthy that MAFA and MAFB are expressed in a distinct manner in mouse and human islet β cells, with only MAFA produced in mouse β cells and MAFA and MAFB in human β cells (26). In fact, misexpression of MAFB is unable to rescue the many deficiencies associated with mice lacking MAFA in β cells (27). These TFs are not often coexpressed in human tissues, the exception being testes, islets, and skeletal muscle (Fig. S8). In these unusual circumstances, MAFA and MAFB presumably act in a heterodimer complex. In our AlphaFold 2 models, heterodimerization ability of MAFA and MAFB variants does not appear to be affected. The degree of transactivation exerted by MAFA and MAFB has been reported to be comparable in β cell culture systems (3), suggesting that transactivation by the heterodimer complex with these MAF variants would be comparable to that of their homodimers. Interestingly, human pancreatic islet β cells exhibit a heterogenous expression pattern for MAFA and MAFB, where β cells coexpressing MAFA and MAFB are more transcriptionally and functionally active than those expressing only one or neither factor (28). MAF/MAFB dimerization status was not evaluated in this study and would warrant further investigation given our results.

## Experimental procedures

### Modeling by AlphaFold 2

Multiple sequence alignments were carried out with MMSeqs2 (29) and AlphaFold 2 models were generated with ColabFold (https://mirdita.de/publication/mirdita-2022) (22, 30) using model parameters version 2.3. To allow for a more robust exploration of conformational space each alignment was run with five seeds leading to 25 models. To examine the effect of phosphorylation on the generated models, we used phosphomimetics based on previously identified phosphorylation sites in MAFA (16). Specifically, serine (S) residues were replaced with aspartic acid (D), and threonine (T) residues were replaced with glutamic acid (E, Fig. 3*B*). These substitutions were made across the multiple sequence alignments excluding gaps. All of the phosphorylation sites in MAFA^WT^ and their homologous sites in MAFB^WT^ were replaced, whereas in conditions that mimic partial phosphorylation, all of the phosphorylation sites were replaced except for residues 49, 53, 57, 61, and 65 in MAFA^S64F^ or residues 54, 58, 62, and 66 in MAFB^S70A^. The highest predicted interface for each of the modelers is shown. To quantitate the variability in the AlphaFold 2 models, distances from Cα for each residue to a fixed point in space were measured. The fixed point was the centroid between R281 in MAFA, MAFA/B, and MAFA/B^T^, R259 in MAFB and R264 in MAFB/A. The distances were averaged for each set of 25 models.

### DNA constructs

The CMV-driven human MAFA and MAFB expression constructs used in the transfections were generated by VectorBuilder Inc (Chicago, IL). DNA sequencing analysis through Genewiz (South Plainfield, NJ) confirmed the fidelity of each construct.

### Cell culture and assays

Min6 and HeLa monolayer cells (31) were maintained in Dulbecco’s modified Eagle medium (Invitrogen) supplemented with 10% and 5% heat inactivated fetal bovine serum, respectively. CMV-driven MAFA, MAFB, PDX1, GLIS3, ISL1, and/or NEUROD1 were cotransfected with the rat II *Insulin* enhancer −238 WT:firefly luciferase plasmid (1), and the phRL-TK Renilla luciferase internal control plasmid. Reporter activity was evaluated 48 h after transfection using a dual-luciferase assay according to the manufacturer’s protocol (Promega). Firefly luciferase measurements were normalized to the phRL-TK activity. Western blotting and gel shift (electrophoretic mobility shift assay, EMSA) reactions were performed as previously described (31) with 10 μg of nuclear extract and 200 fmol of the biotin-labeled double-stranded human *INS* MAFA/B binding site probe or MARE probes (12) mixed either alone, with unlabeled competitor DNAs, MAFA antibody (Bethyl Laboratories, Product # A700–067), or MAFB antibody (Bethyl Laboratories, Product # A700–046) in a 20 μl reaction system (LightShift Chemiluminescent EMSA Kit, Thermo Fisher Scientific) containing 1x binding buffer, 2.5% glycerol, 5 mM MgCl, 50 ng/μl of poly(dI-dC) (Thermo Fisher Scientific), and 0.05% NP-40 (Sigma-Aldrich). The WT and mutant MAFA/B binding site sequences were described previously (31). The reactions were separated on a 6% precast DNA retardation gel in 0.5% Tris borate-EDTA buffer (TBE, Thermo Fisher Scientific) at 100 V for 1.5 h. Each experiment was repeated at least three times using independently isolated protein preparations.

### Statistical analysis

Data are expressed as the mean ± SEM. Statistical analysis was performed using GraphPad Prism 10.2.2 (GraphPad Software Inc; https://graphpad.com). The difference between groups were analyzed by mixed-effects analysis or 2-way ANOVA followed by either Dunnett’s or Tukey’s multiple comparisons tests as indicated in the figure legends. Differences were considered to be statistically significant when *p* < 0.05.

## Data availability

The data generated in this study are within the article or in the Supporting Information files.

## Supporting information

This article contains supporting information (32, 33).

## Conflict of interest

The authors declare that they have no conflicts of interest with the contents of this article.
