# Supplementary material for: Defining unique structural features in the MAFA and MAFB transcription factors that control Insulin gene activity
Source: J Biol Chem. 2024 Oct 28;300(12):107938. doi: 10.1016/j.jbc.2024.107938 (PMC11626809; doi:10.1016/j.jbc.2024.107938)
Supplement: Supporting Figure Legends [file mmc1.docx]

**Supplemental Figure 1. Overlay of MAFA and MAFB crystal structures.** The structures were aligned based on the DNA used in MAFA (4EOT, red) (Pogenberg et al., 2014) and MAFB (2WTY, blue) (Lu et al., 2012c) structural analysis. The top panel shows both the protein and DNA, while the bottom panel has the DNA removed to highlight the residues involved in DNA binding. A closer view of a subset of these residues is shown in **Figure 1B**.

**Supplemental Figure 2. The MAFB basic region mutant protein was produced at WT-like levels.** MAFB (Bethyl Laboratories, Product # A700-046) immunoblot analysis was performed on the transfected nuclear proteins used the gel shift assays.

**Supplemental Figure 3. AlphaFold 2 modeling of the full-length MAFA and MAFB proteins.** A) Variant proteins MAFA^S64F^ or MAFB^S70A^ prevent subsequent GSK3-mediated phosphorylation (Rocques et al., 2007). B-C) The structure of models developed by AlphaFold 2 for MAFA^un^ and MAFB^un^ (left), MAFA^WT^ and MAFB^WT^ (center), and MAFA^S64F^ and MAFB^S70A^ (right). D) Plots of the mean distance to a fixed point in space for MAFA^un^, MAFA^WT^, and MAFA^S64F^ (left) and MAFB^un^, MAFB^WT^, and MAFA^S70A^ (right). The fixed point is the centroid between arginine (R) 281 in MAFA and R259 in MAFB.

**Supplemental Figure 4. MAF mutants retain dimerization capacity but impact position of protein regions.** A) Top ranked model for each of the 5 AlphaFold modellers. There is little variation in the orientation of the two L-Zip domains in the homodimers. For the three heterodimers there is some variation in the orientation of the L-Zip domains. The N-terminal regions prior to the L-Zip domains have been removed for clarity. B) Plots of the mean distance to a fixed point in space (centroid) for each set of models. Left, distances for MAFA within the three heterodimers compared to the MAFA homodimer. Right, distances for MAFB within the three heterodimers compared to the MAFB homodimer. The fixed point is the centroid between R272 in MAFA and R256 in MAFB.

**Supplemental Figure 5**. **MAF and their variants exhibit similar binding properties**. A) Gel shift assays of HeLa nuclear extract produced MAFA^WT^ and mutant MAFA^S64F^ bound to MARE consensus sequence and to the MARE Mutant probes (Left, TGC and Right, CEN). Specificity of binding was interrogated by unlabeled competitor probes in excess. B) Gel shift assays of HeLa nuclear extract produced MAFB^WT^ and mutant MAFB^S70A^ bound to MARE consensus sequence and to the MARE Mutant probes (Left, TGC and Right, CEN). MAFA^WT^ and MAFA^S64F^ were super-shifted (SS) upon addition of MAFA antibody, while MAFB^WT^ and MAFB^S70A^ were super-shifted (SS) upon addition of MAFB antibody. C) Sequences of probes used in EMSA. Mutations from the consensus 13bp MARE sequence indicated in red.

**Supplemental Figure 6. Basal transcription factor expression and NEUROD1 production in HeLa transfected cells.** A) Basal expression of HeLa-enriched transcription factor genes, *BCL6* and *MKRN1*, and cytokeratin *CK19* compared with the expression of islet-enriched factors. B) Transfected NEUROD1 protein levels were determined by immunoblotting using anti-NEUROD1 antibodies (Invitrogen , Product # JM11-10). A representative blot is shown. n=3.

**Supplemental Figure 7. AlphaFold 2 modeling of the WT and mutant MAFA and MAFB chimeras.**  A) MAFA^WT^, MAFA^WT^/B, MAFA^S64F^/B, and MAFA^WT^/B^T^. B) MAFB^WT^, MAFB^WT^/A, and MAFB^S70F^/A. C) Plots of the mean distance to a fixed point in space for each set of models. The fixed point is the centroid between R281 in MAFA and MAFA/B chimeras, R259 for MAFB, and R264 for the MAFB/A chimeras.

**Supplemental Figure 8. MAFA and MAFB are only co-expressed in testis, skeletal muscle and pancreatic islets in humans.**  The mRNA expression levels of MAFA and MAFB across different human tissues were queried in A) the Genotype-Tissue Expression (GTEx) project. The testis and skeletal muscle data are highlighted in red circle (Lonsdale et al., 2013). B) Translational human pancreatic islet genotype tissue-expression resource (TIGER) for MAFA and MAFB (Alonso et al., 2021). TPM: transcript per million.
