# Supplementary material for: Defining unique structural features in the MAFA and MAFB transcription factors that control Insulin gene activity
Source: J Biol Chem. 2024 Oct 28;300(12):107938. doi: 10.1016/j.jbc.2024.107938 (PMC11626809; doi:10.1016/j.jbc.2024.107938)
Supplement: Supporting Figures [file mmc2.pdf]

Structures of MAFA overlaid on MAFB

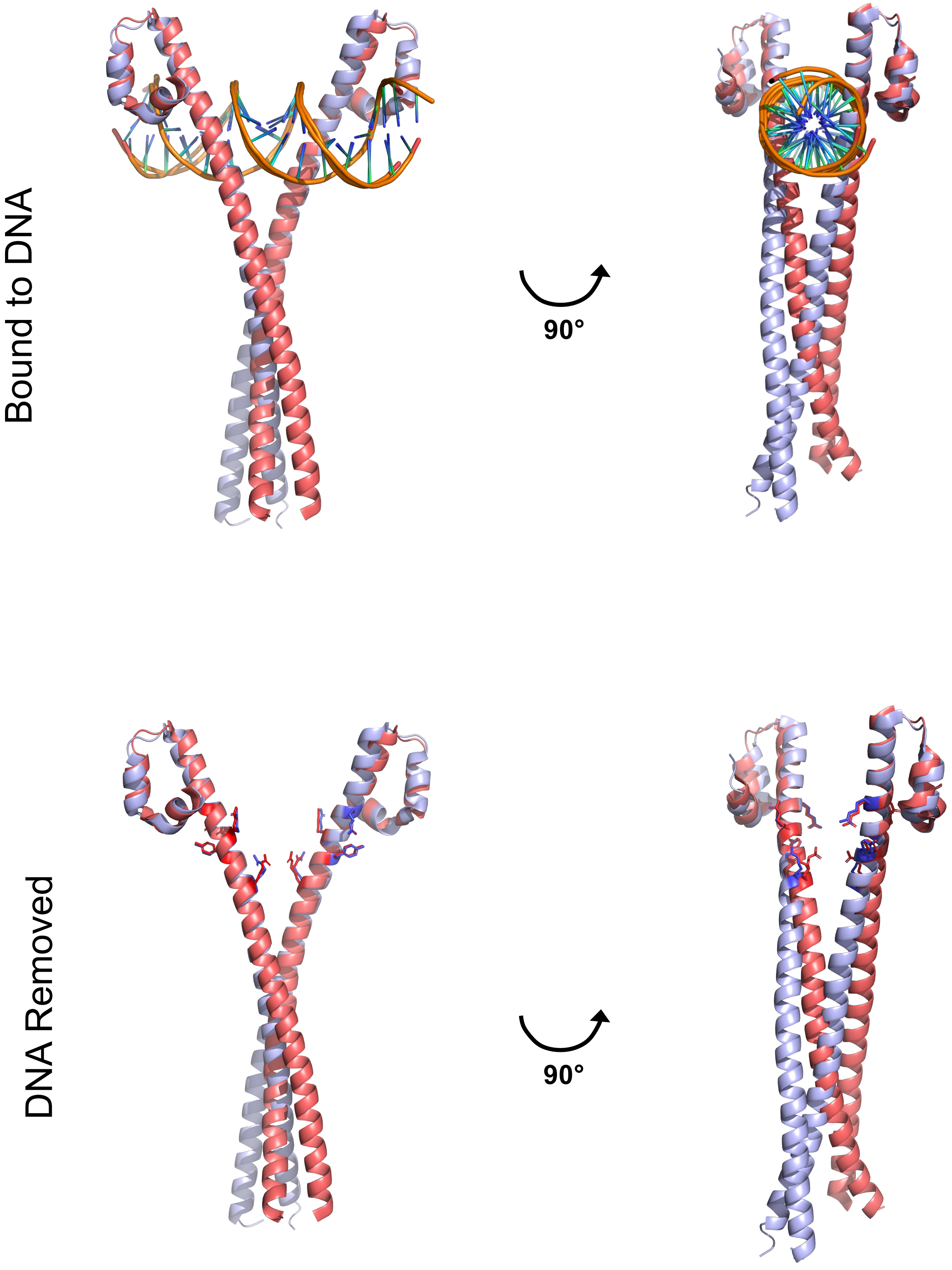

Supplemental Figure 1

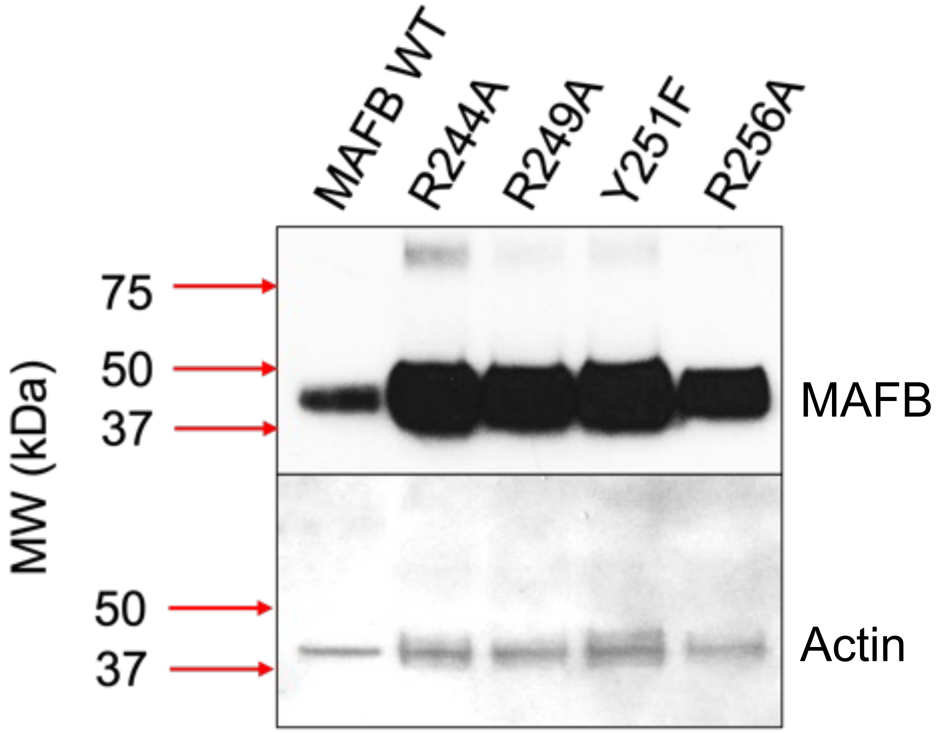

Supplemental Figure 2

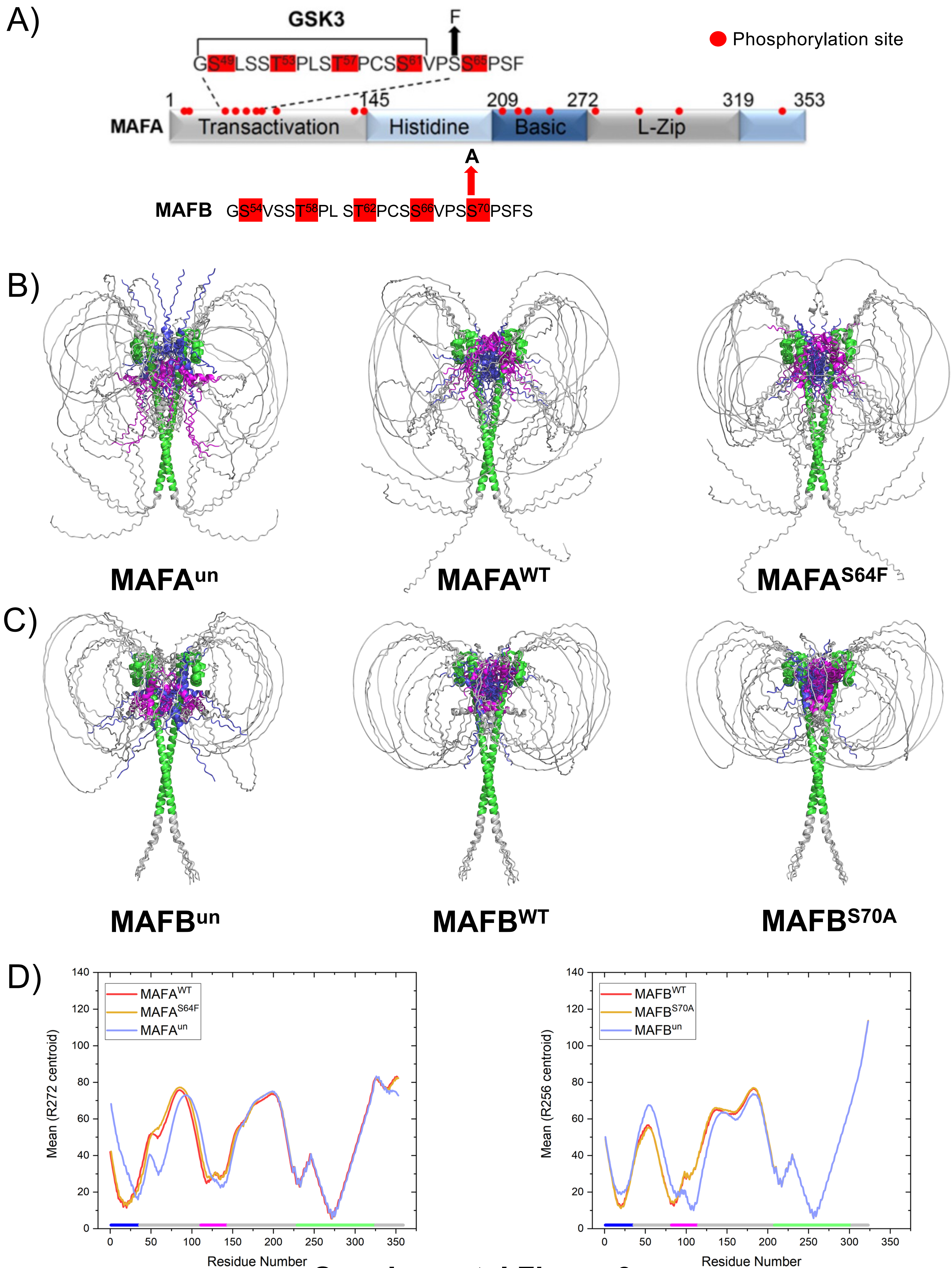

Supplemental Figure 3

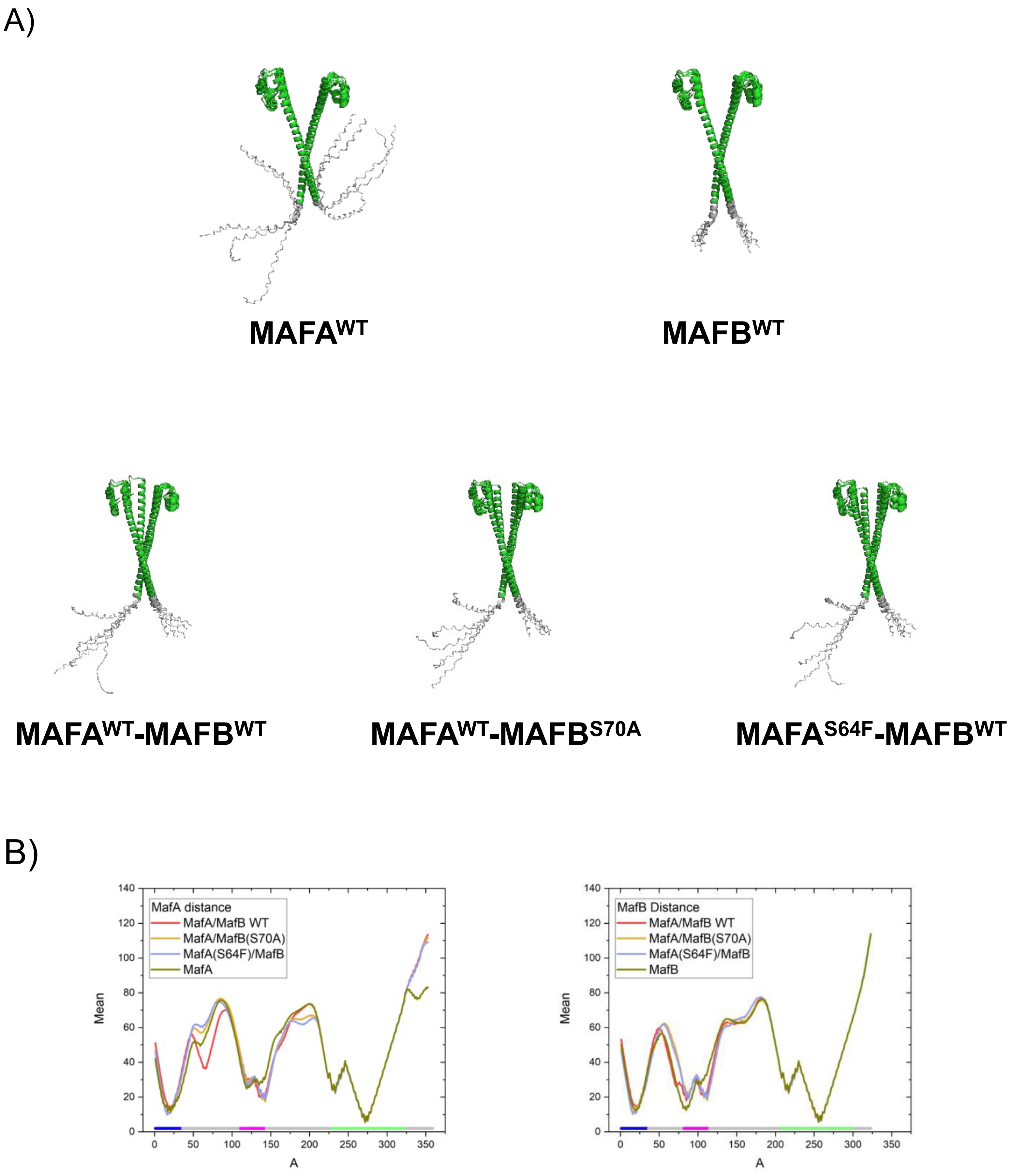

Supplemental Figure 4

A)

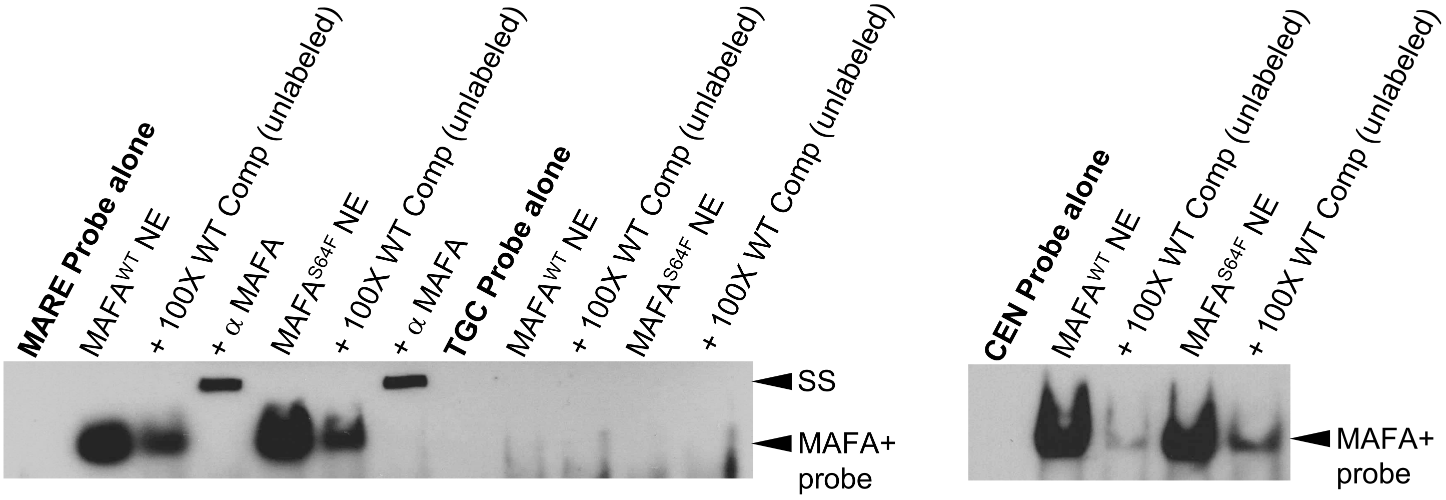

B)

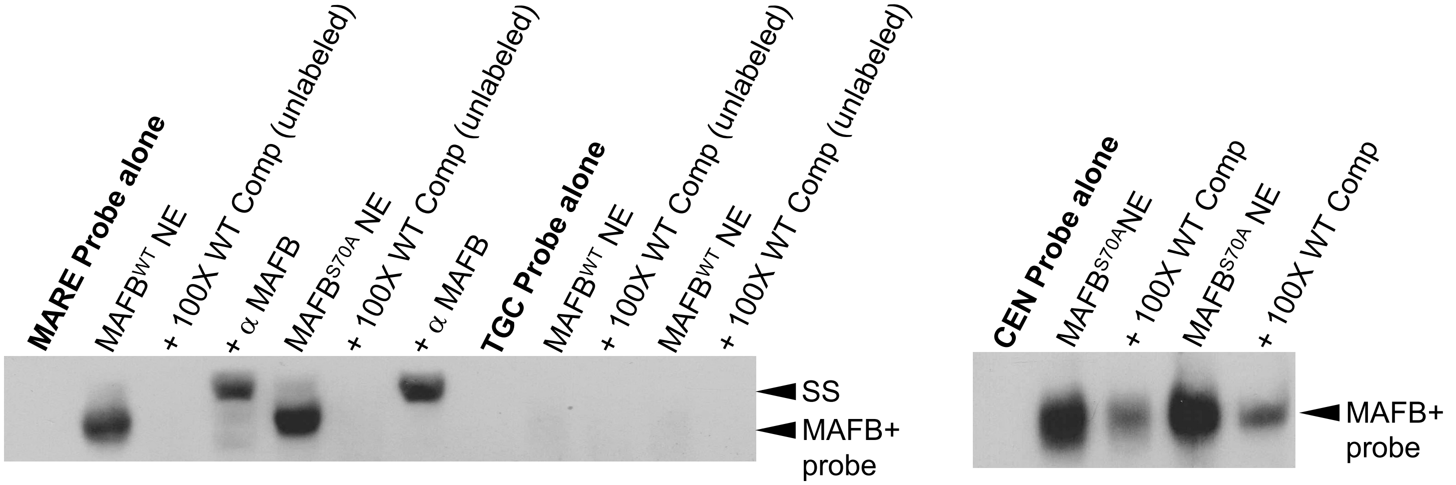

C)

Probe	Sequence
INS	CAGGCCC GGAAAT TGC AGC C TCA GCC GCGCGA CCTGTAC
MARE	CAGGCCC CGCCCG TGC TGA C TCA GCA GCGCGA CCTGTAC
Mut_MARE	CAGGCCC CGCCCG TAT TGA T TCA TAA GCGCGA CCTGTAC
TGC	CAGGCCC CGCCCG AAT TGA C TCA GCA GCGCGA CCTGTAC
CEN	CAGGCCC CGCCCG TGC TGA T TCA GCA GCGCGA CCTGTAC

Supplemental Figure 5

A)

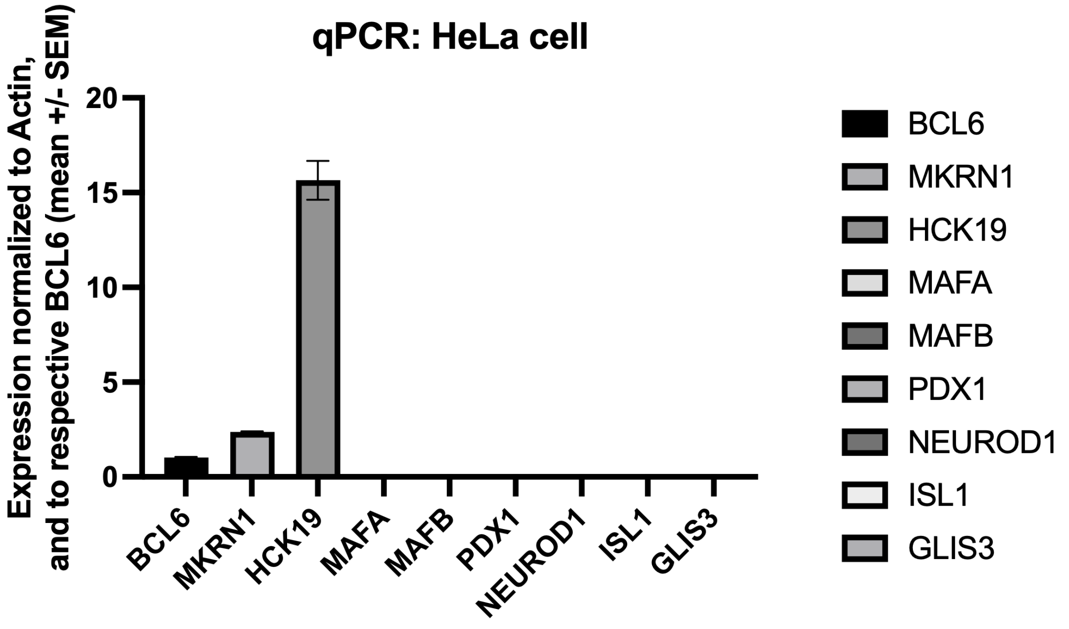

B)

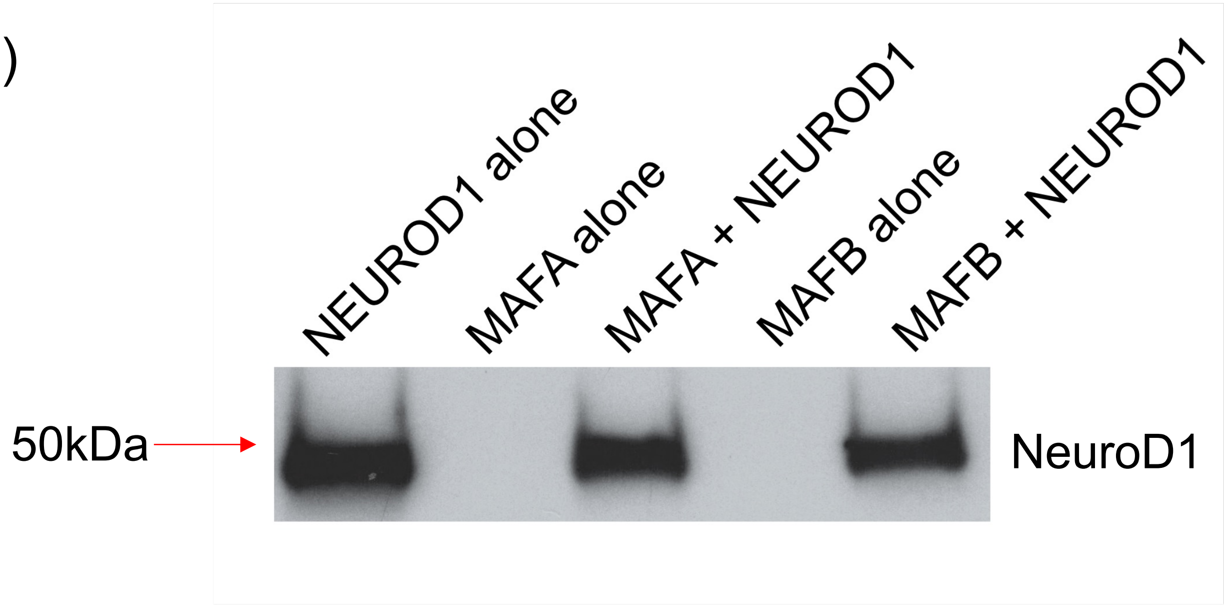

Supplemental Figure 6

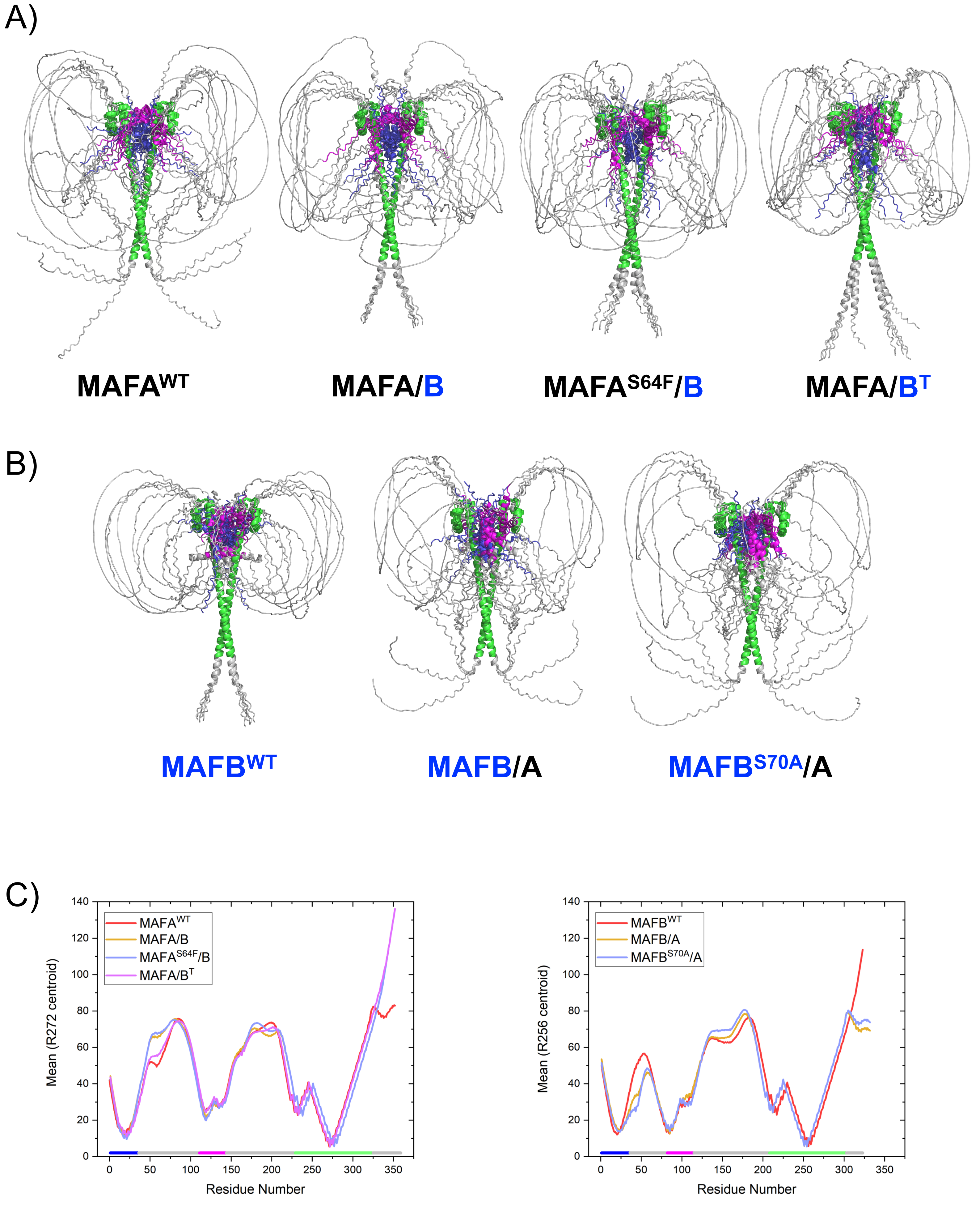

Supplemental Figure 7

A)

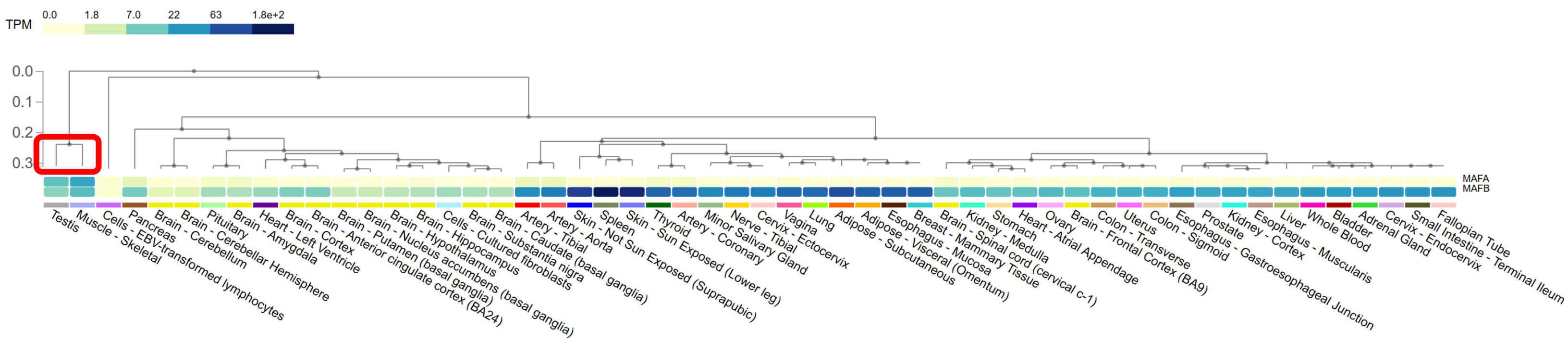

B)

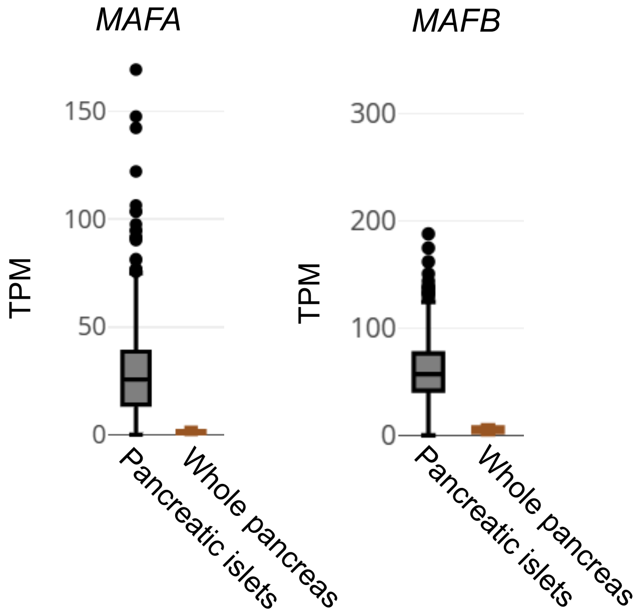

Supplemental Figure 8
